# A107 WHAT SIZE CUT-OFF LEVEL SHOULD BE USED TO IMPLEMENT OPTICAL POLYP DIAGNOSIS?

**DOI:** 10.1093/jcag/gwab049.106

**Published:** 2022-02-21

**Authors:** M Taghiakbari, H Pohl, R Djinbachian, J Anderson, D Metellus, A N Barkun, M Bouin, D von Renteln

**Affiliations:** 1 Centre Hospitalier de l’Universite de Montreal, Longueuil, QC, Canada; 2 Dartmouth Geisel School of Medicine, Hanover, NH; 3 Gastroenterology, McGill University, The Montreal General Hospital, GI Division, Montreal, QC, Canada

## Abstract

**Background:**

The risk of advanced pathology and potential mismanagement increases with polyp size while performing optical diagnosis. We hypothesized that a lower polyp size cut-off (e.g., 1–3 mm) would be associated with a lower risk of misclassifying advanced neoplasia or even cancer when using optical diagnosis.

**Aims:**

This study aimed to evaluate the proportion of patients undergoing inadequate surveillance intervals associated with different size cut-offs when using optical diagnosis.

**Methods:**

In a post-hoc analysis of three prospective studies, the use of optical diagnosis was evaluated for three polyp size groups: 1–3, 1–5, and 1–10 mm. The primary outcome was the proportion of patients in which advanced adenomas were found and optical diagnosis resulted in delayed surveillance in each group. Secondary outcomes included agreements between surveillance intervals based on high-confidence optical diagnosis and pathology outcomes, reduction in histopathological examinations, and proportion of patients who could receive an immediate surveillance recommendation.

**Results:**

We included 3374 patients (7291 polyps ≤10 mm) undergoing complete colonoscopies (median age 66.0 years, 75.2% male, 29.6% for screening). Among polyp sized 1–3 mm, 1–5 mm, and 1–10 mm, 0.5%, 0.6%, and 1.2% of polyps, respectively, were found to have advanced pathology ( *P <.0001*). The percentage of patients with advanced adenomas and either 2- or 7- year delayed surveillance intervals (n=79) was 3.8%, 15.2%, and 25.3% for size cut-offs of 1–3, 1–5, and 1–10 mm polyps, respectively ( *P*<.0001). Surveillance interval agreements between pathology and high-confidence optical diagnosis for the three groups were 97.2%, 95.5%, and 94.2%, respectively. In the cohort of patients in which patients with normal colonoscopy, polyps >10 mm, and poor bowel preparation were excluded, the surveillance interval agreements between pathology and high-confidence optical diagnosis for the three groups were 96.2%, 93.6%, and 92.1%, respectively. Total reduction in pathology examinations for the three groups were 33.5%, 62.3%, and 78.2%, respectively. Furthermore, optical diagnosis could have allowed 41.0%, 58.2%, and 73.3% of patients, respectively, to be given immediate surveillance interval recommendations.

**Conclusions:**

This study showed that limiting optical diagnosis to polyps 1–3 mm resulted in an excellent safety profile with a very low risk for inappropriate management of advanced adenomas, which makes routine clinical implementation of the “resect and discard” strategy feasible. Implementing a 3 mm cut-off could be a starting point for endoscopists to feel comfortable with the “resect and discard” strategy, with the potential of implementing a 5 mm cut-off, once optical diagnosis becomes more popular, and endoscopists become more comfortable with its use.

**Funding Agencies:**

NoneNA

**Figure:**
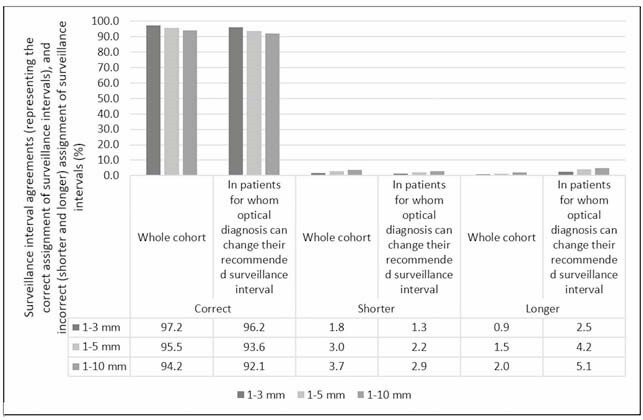
**Figure 1.** The percentage of surveillance interval agreement (correct assignment of surveillance intervals) between histopathology and optical diagnosis in all patients with valid determination of surveillance intervals, and in patients for whom optical diagnosis could have affected their next recommended surveillance intervals, using the NICE classification.

